# Scientific Items

**Published:** 1878-11-20

**Authors:** 


### *Scientific Items.*

### ILLUSIONS.

The natural world is full of illusions. The apparent rising and setting of the sun, the gorgeous clouds that proves to be only a dreary mist when you get caught in them, the mirage, that reveals things lying below the horizon, and shows us ships sailing keel up in the air, the full moon, which, as it emerges from the horizon, appears to be twice as large as it does when it is over our heads, while, if looked at thorugh a tube or measured by an instrument, is found to be of precisely the same diamter, the coming together to a point of two right lines when seen in prospective, the mistake of supposing the train in which we are seated to be in motion when another train at our side begins to start, the deceptive idea that we have of distance, as in the instance of loftymountians, which may seem to be close at hand, when, in fact, it is scores of miles away; these are illusions of sight that are familiar to us all. There are other forms of optical illusion, which depend upon the principle that motion may be quicker than sight, such as the extraordinary tricks of the juggler, or prestidigitator, which is now the favorite title of the professors of this science, the continuous circle of fire produced by whirling a lighted stick in the air, and the fantastic movement of painted figures in the popular toy known as zootrope. And again, the most marvelous illusions may be the result of an excited fancy, as when one sees specters and hobgoblins.

### BALOONING SPIDERS.

The Rev. H. C. McCook, of Philadelphia, describing the balooning habit, or flight of spiders, says the spider seeks a high position, as the top of a fence-post, as the point of ascent. The abdomen is elevated to as nearly a right angle with the thorax as may be; a pencil of threads issues from the spinnerets, the face being meanwhile turned to various points until it looks in the direction of the wind. The legs are then stretched upward, thus raising the body aloft, and the insect gradually assumes a position as if resisting some force from above. Suddenly the right claws are unlossed, the spider mounts with a sharp bound and floats off, generally with the back downward, but sometimes with the position reversed. At first the abdomen seems to be in advance, but generally the body is turned so that the head is front. The pencil of threads is caught by the feet, and floats out in front. Upon these threads the spider will climb upward as though to adjust the centre of gravity. Meanwhile a pencil of threads floats out behind, leaving the spider to ride in the angle of the two, or sometimes three pencils. The feet seem to be united by delicate filaments, which serve to increase the buoyancy of the balloon. The insect is carried forward by the wind, riding for long distances in an open space, and often high up upon ascending currents. Its anchorage appears at times to be within its own volition, by drawing in with the claws the forward pencil and gathering it in a white roll within the mandiblet; but most frequently the progress of the insect is stopped by some elevated object, or by the subsidence of the breeze.

---

The Journal of Mocroscopy says the entire Bible could be photographed on a little less than an inch and a half. It could be photograped, nearly ten times on an ordinary postal card!

